# Supplementary material for: ILIAD: a suite of automated Snakemake workflows for processing genomic data for downstream applications
Source: BMC Bioinformatics. 2023 Nov 8;24:424. doi: 10.1186/s12859-023-05548-x (PMC10633908; doi:10.1186/s12859-023-05548-x)
Supplement: Supplementary file 1 — Additional file 1. Supplementary tables and figures. [file 12859_2023_5548_MOESM1_ESM.pdf]

ILIAD: A suite of automated Snakemake workflows for processing genomic data for downstream applications

Supplementary Material

Herrick, N. Walsh, S.

Table S1. Time and resource usage for *Iliad*'s raw sequence read data module. Each rule in the Snakemake workflow utilized the "benchmark" directive to collect this data. Some rules will constitute a number of jobs depending on the wildcards e.g. chromosomes and number of splits per chromosome. For this reason, values for time and resource usage were averaged across the jobs per rule. The benchmarks were conducted with paired-end reads of one sample, KPGP-00127, from the open-source Korea Personal Genome Project (KPGP: http://kpgp.kr) on a dedicated remote server in the Department of Biology at Indiana University – Purdue University Indianapolis. This run was conducted using 8 cores provided to Snakemake for 8 jobs in parallel. No internal rule parallelization occurred; however, it is possible and greatly speeds up some rules e.g. alignment. Since the "benchmark" directive is rule-specific for resource monitoring, there was no impact on any independent rule's time and resource report from performing these benchmarks with 8 jobs in parallel.

Rule	Jobs (with 5 splits)	Average values across Jobs per Rule								
		h:m:s	max_rss	max_vms	max_uss	max_pss	io_in	io_out	mean_load	cpu_time
alignment	1	2:02:25	19083.97	20901.21	19071.88	19075.59	12.76	42668.80	1186.09	232.43
annotate_rsIDs_with_dbSNP	23	0:20:44	70.60	395.50	61.68	64.75	11.64	54.34	98.54	1226.49
annotation_index	23	0:00:02	3.36	6.99	3.44	3.44	2.63	0.00	62.19	1.99
bwa_index	1	0:51:34	4477.62	4486.36	4469.03	4471.97	10.88	6651.68	99.17	3069.47
cat_splits_to_chr_list	23	< 0:00:01	0	0	0	0	0	0	0	0
check_for_chr_string	1	0:00:01	24.41	28.84	21.45	23.00	8.98	0	0	0.42
concat_reads	1	0:05:51	4.37	14.71	2.28	3.37	2.64	49940.04	88.06	167.87
concat_splits_per_chrom	23	0:00:19	1462.44	7012.67	1452.97	1456.09	11.66	448.54	87.93	10.68
concat_splits_per_chrom_index	23	0:00:02	3.31	6.95	3.41	3.41	2.40	0	57.14	1.88
download_NYGC_annotations_file	23	1:23:16	4.31	9.35	4.38	4.38	3.17	3092.38	1.76	89.07
download_dbSNP_file	1	0:07:16	8.79	13.42	6.49	7.95	4.23	15607.06	33.24	0.09
fnPath_to_file	115	< 0:00:01	0	0	0	0	0	0	0	0
get_genome	1	0:04:44	53.21	1362.86	41.79	45.18	16.06	2380.64	13.57	38.46
merge_sample_chrom_list	23	< 0:00:01	23.15	418.16	20.82	21.89	8.42	0	0	0.39
merge_samples_per_chrom	23	0:00:25	44.25	223.59	35.42	38.49	11.50	36.50	67.21	15.09
merge_samples_per_chrom_index	23	0:00:02	3.33	6.98	3.42	3.42	2.52	0	60.35	1.93
split_chroms	115	< 0:00:01	0.51	2.62	1.54	1.54	2.03	30.22	0	0.15
split_chroms_chr_string	115	< 0:00:01	0.55	2.62	1.56	1.56	2.04	69.42	0	0.25
tabbed_regions	23	0:00:02	41.75	450.92	33.03	36.49	9.38	42.65	103.49	2.94
tabbed_regions_chr_string	23	0:00:02	40.72	339.76	32.91	36.03	10.71	64.81	67.59	2.74
unique_regions_file	23	0:00:11	4.70	10.25	2.37	3.79	1.98	67.80	99.26	11.62
variant_calling	115	0:21:30	231.96	347.63	218.21	222.68	11.80	10.83	99.51	1289.78
variant_calling_index	115	< 0:00:01	3.19	36.91	3.38	3.38	2.67	0.00	3.05	0.43
wget_reads_from_url	1	0:16:59	4.39	9.35	4.41	4.41	3.17	17565.03	18.27	186.41

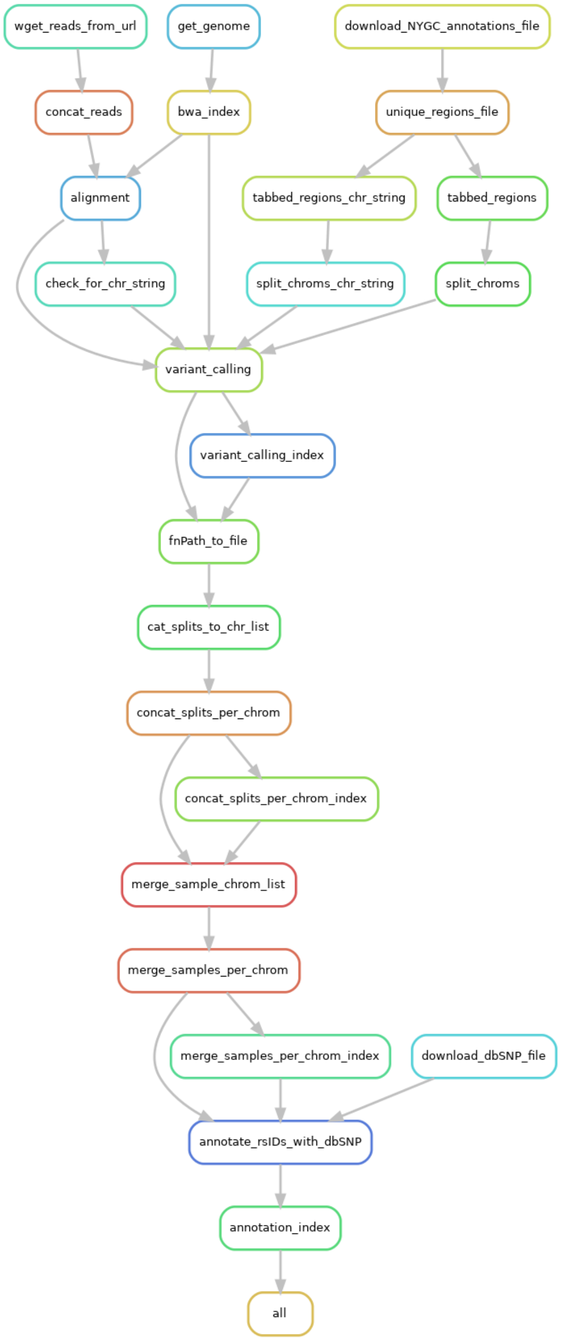

Figure S1. A directed acyclic graph for Iliad's raw sequence read data module.

Table S2. Time and resource usage for *Iliad*'s stored sequence read data module. Each rule in the Snakemake workflow utilized the "benchmark" directive to collect this data. Some rules will constitute a number of jobs depending on the wildcards e.g. chromosomes and number of splits per chromosome. For this reason, values for time and resource usage were averaged across the jobs per rule. The benchmarks were conducted on one sample, NA12718, in CRAM format from the 1000 Genomes Project (ftp://ftp.sra.ebi.ac.uk/vol1/run/ERR323/ERR3239480) on a dedicated remote server in the Department of Biology at Indiana University – Purdue University Indianapolis. This run was conducted using 16 cores provided to Snakemake for 16 jobs in parallel. No internal rule parallelization occurred. Since the "benchmark" directive is rule-specific for resource monitoring, there was no impact on any independent rule's time and resource report from performing these benchmarks with 16 jobs in parallel.

Rule	Jobs (with 5 splits)	Average values across Jobs per Rule								
		h:m:s	max_rss	max_vms	max_uss	max_pss	io_in	io_out	mean_load	cpu_time
annotate_rsIDs_with_dbSNP	23	0:31:40	73.66	666.09	64.91	67.97	103.95	68.71	99.18	1885.39
annotation_index	23	0:00:04	2.39	4.98	2.47	2.47	2.14	0.01	25.75	2.66
bwa_index	1	-	-	-	-	-	-	-	-	-
cat_splits_to_chr_list	23	< 0:00:01	11.84	846.32	10.90	10.91	0.00	0.00	0.00	0.01
check_for_chr_string	1	0:00:01	36.50	1434.71	33.45	34.97	21.24	0.10	35.07	0.80
concat_splits_per_chrom	23	0:00:35	1533.85	6999.38	1524.93	1528.00	23.09	366.06	78.72	16.36
concat_splits_per_chrom_index	23	0:00:03	12.43	793.84	11.77	11.78	2.97	0.00	72.20	2.96
cram_variant_calling	115	5:46:43	585.14	2038.99	568.49	573.08	136.39	120.02	99.86	16570.40
variant_calling_index	115	0:00:01	8.84	440.94	8.48	8.48	3.19	0.02	40.11	0.58
download_NYGC_annotations_file	23	-	-	-	-	-	-	-	-	-
download_dbSNP_file	1	-	-	-	-	-	-	-	-	-
fnPath_to_file	115	< 0:00:01	23.92	1548.80	22.14	22.16	0.00	0.00	0.00	0.03
get_genome	1	-	-	-	-	-	-	-	-	-
merge_sample_chrom_list	23	0:00:01	23.24	660.61	20.77	21.73	12.39	0.00	5.13	0.37
merge_samples_per_chrom	23	0:00:51	47.64	509.40	38.99	42.01	19.36	49.58	66.33	36.76
merge_samples_per_chrom_index	23	0:00:03	18.66	1156.11	17.23	17.25	3.25	0.07	71.78	3.33
rename_cram	1	< 0:00:01	0.00	0.00	0.00	0.00	0.00	0.00	0.00	0.00
split_chroms	115	-	-	-	-	-	-	-	-	-
split_chroms_chr_string	115	-	-	-	-	-	-	-	-	-
tabbed_regions	23	-	-	-	-	-	-	-	-	-
tabbed_regions_chr_string	23	-	-	-	-	-	-	-	-	-
unique_regions_file	23	-	-	-	-	-	-	-	-	-
wget_cram	1	1:19:52	4.48	9.36	4.48	4.48	3.94	14673.86	4.99	238.90

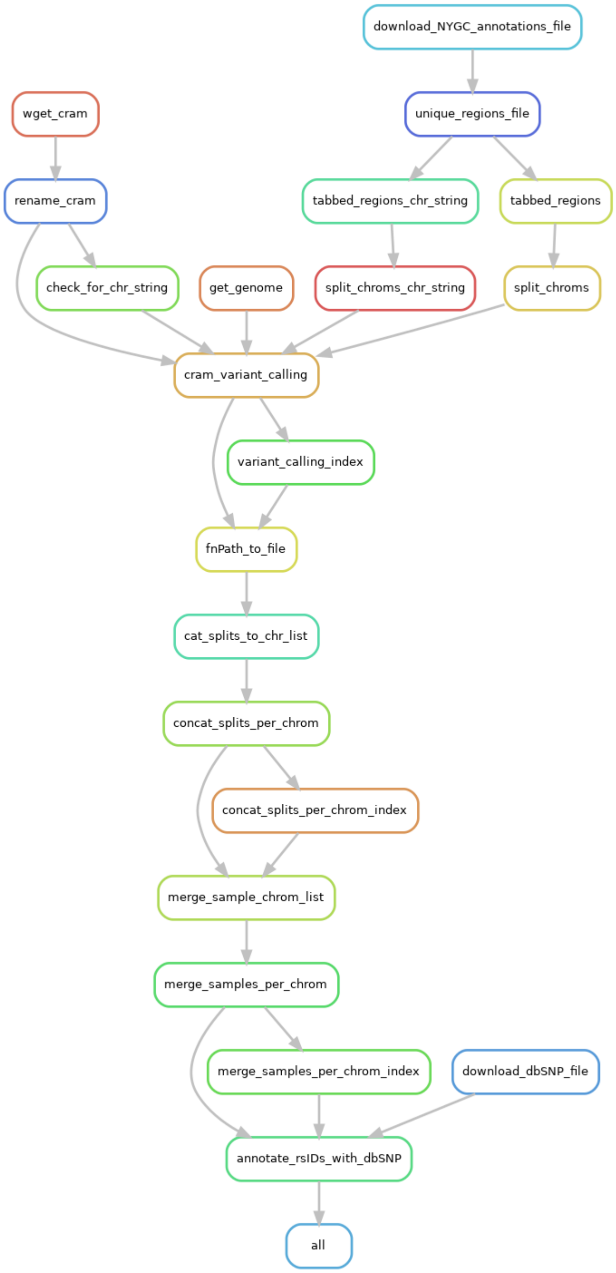

Figure S2. A directed acyclic graph for Iliad's stored sequence read data module.

Table S3. Time and resource usage for *Iliad*'s SNP Array data module. Each rule in the Snakemake workflow utilized the "benchmark" directive to collect this data. Some rules will constitute a number of jobs depending on the wildcards e.g. chromosomes and number of splits per chromosome. For this reason, values for time and resource usage were averaged across the jobs per rule. The benchmarks were conducted on three in-house IDAT samples on a dedicated remote server in the Department of Biology at Indiana University – Purdue University Indianapolis. This run was conducted using 8 cores provided to Snakemake for 8 jobs in parallel. No internal rule parallelization occurred. Since the "benchmark" directive is rule-specific for resource monitoring, there was no impact on any independent rule's time and resource report from performing these benchmarks with 8 jobs in parallel.

Rule	Jobs	Average values across Jobs per Rule								
		h:m:s	max_rss	max_vms	max_uss	max_pss	io_in	io_out	mean_load	cpu_time
bwa_index	1	-	-	-	-	-	-	-	-	-
cleanRegionsFile	1	0:00:22	2.14	45.77	1.65	1.69	0	0	0	0
download_MEGA_physical_genetic_coordinates	1	0:01:03	27.53	1571.78	27.06	27.08	0.07	0	0	0.03
download_dbSNP_file	1	-	-	-	-	-	-	-	-	-
download_iaap	1	0:00:15	0.02	1.22	0.03	0.03	0	0	0	0
download_lociNames_conversion_file	1	0:01:02	20.06	730.39	15.18	15.25	0	0	0	0.02
download_product_files	1	0:02:23	7.75	45.77	7.25	7.32	0	0	0	0
extract_clean_regions	1	0:10:44	28.54	755.38	27.9	27.93	0	0	0	0.02
filter_for_dbSNP_vars	1	0:21:10	0.02	1.22	0.03	0.03	0	0	0	0
gencall	1	0:01:34	8.27	45.77	7.86	7.93	0	0	0	0.01
GenTrain_ClusterSep_QC	1	0:00:12	21.78	742.04	21.13	21.19	0	0	0	0.01
get_genome	1	-	-	-	-	-	-	-	-	-
gtc2vcf	1	0:03:50	6.53	45.77	6.16	6.21	0	0	0	0.01
insert_rsIDS	1	0:00:18	6.7	45.77	0.02	0.02	0	0	0	0
original_lociNames	1	0:00:03	0.02	1.22	0.03	0.03	0	0	0	0
replace_lociNames_to_rsIDs	1	0:00:39	9.32	45.77	8.88	8.95	0	0	0	0.01

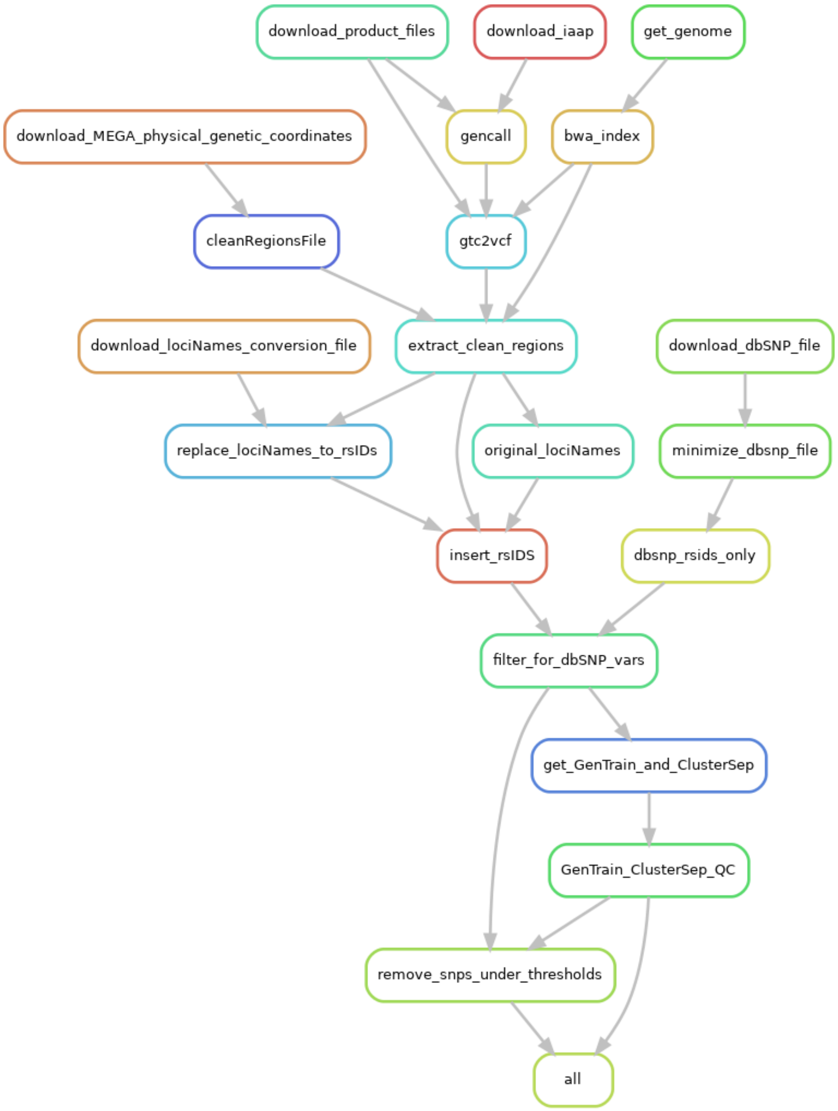

Figure S3. A directed acyclic graph for Iliad's SNP Array data module.

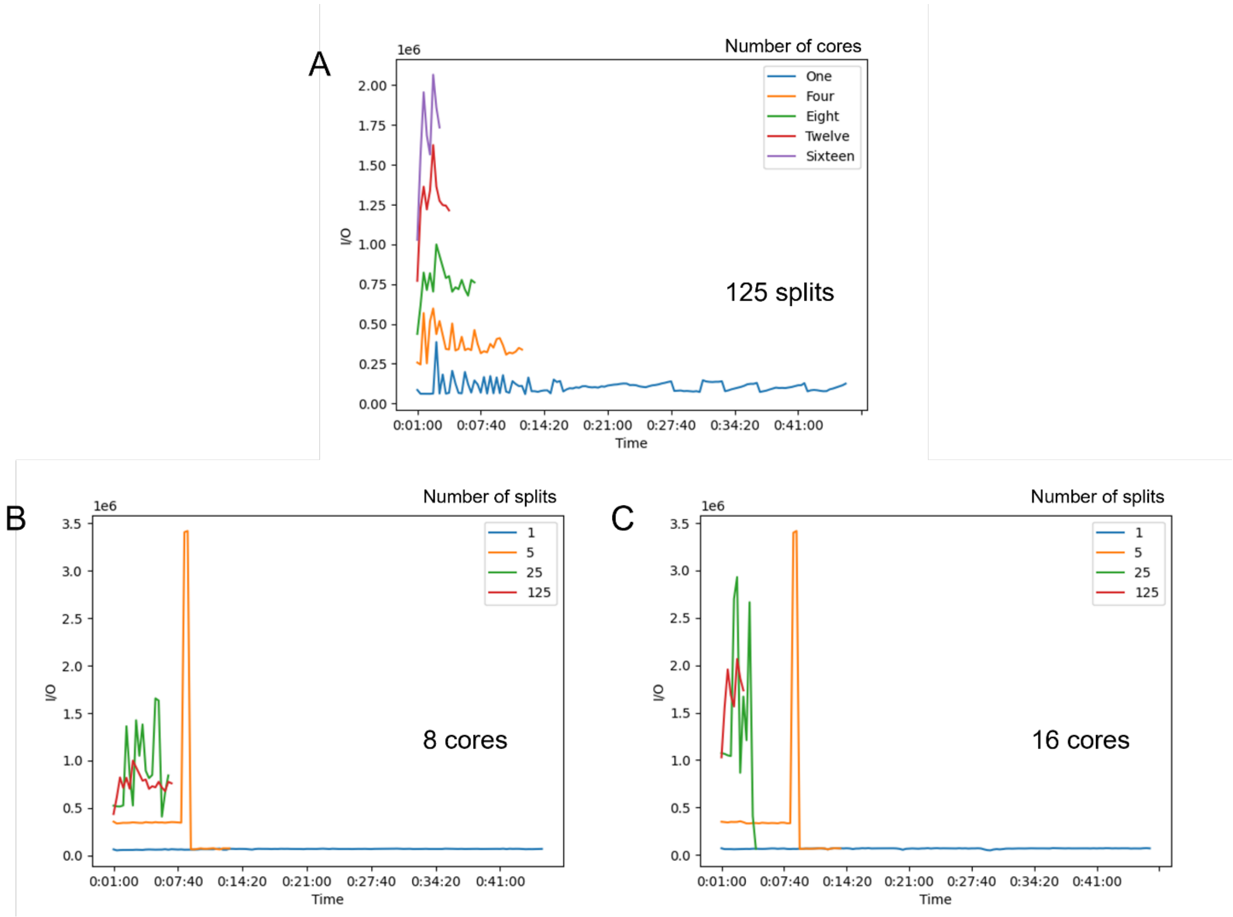

Figure S4. Time (h:m:s) and I/O (write data in KB) metrics for variant calling of one sample on chromosome 22 at **A)** one, four, eight, twelve, and sixteen cores to validate the logic that a large numbers of splits per chromosome, e.g. 125, performed faster with more supplied cores. Performance metrics for a varying number of splits at **B)** eight and **C)** sixteen cores to depict the lack of advantage of high splits with a lower number cores.
